# Does population screening for *Chlamydia trachomatis *raise anxiety among those tested? Findings from a population based chlamydia screening study

**DOI:** 10.1186/1471-2458-6-106

**Published:** 2006-04-25

**Authors:** Rona Campbell, Nicola Mills, Emma Sanford, Anna Graham, Nicola Low, Tim J Peters

**Affiliations:** 1 Department of Social Medicine, University of Bristol, Canynge Hall, Whiteladies Road, BRISTOL BS8 2PR, UK; 2 Bedfordshire and Hertfordshire Strategic Health Authority, 63-77 Victoria Street, St Albans, Herts. AL1 3ER, UK; 3 Department of Community Based Medicine, University of Bristol, 1 The Grange, Woodland Road, Bristol, BS8 1AU, UK; 4 Department of Social and Preventive Medicine, University of Berne, Finkenhubelweg 11, 3012 Berne, Switzerland

## Abstract

**Background:**

The advent of urine testing for *Chlamydia trachomatis *has raised the possibility of large-scale screening for this sexually transmitted infection, which is now the most common in the United Kingdom. The purpose of this study was to investigate the effect of an invitation to be screened for chlamydia and of receiving a negative result on levels of anxiety, depression and self-esteem.

**Methods:**

19,773 men and women aged 16 to 39 years, selected at random from 27 general practices in two large city areas (Bristol and Birmingham) were invited by post to send home-collected urine samples or vulvo-vaginal swabs for chlamydia testing. Questionnaires enquiring about anxiety, depression and self-esteem were sent to random samples of those offered screening: one month before the dispatch of invitations; when participants returned samples; and after receiving a negative result.

**Results:**

Home screening was associated with an overall reduction in anxiety scores. An invitation to participate did not increase anxiety levels. Anxiety scores in men were lower after receiving the invitation than at baseline. Amongst women anxiety was reduced after receipt of negative test results. Neither depression nor self-esteem scores were affected by screening.

**Conclusion:**

Postal screening for chlamydia does not appear to have a negative impact on overall psychological well-being and can lead to a decrease in anxiety levels among respondents. There is, however, a clear difference between men and women in when this reduction occurs.

## Background

Health care is usually provided when an individual patient seeks help. In contrast population-screening programmes seek to identify disease in groups of people who have not sought care. While the overall aim of screening is to improve population health by reducing preventable morbidity and mortality, not everyone in the population screened will benefit as individuals. In particular, screening programmes therefore have the potential to harm by causing unnecessary distress to those who do not have the condition, or by falsely reassuring those who have the condition but who have negative screening tests. Those who test positive and do have the condition have to cope with an unexpected and unsought diagnosis. The costs of screening programmes are usually evaluated in economic terms, but there is increasing recognition of the need to assess the psychological costs for those participating [1,2].

While other conditions such as human papilloma virus infections are considered to be more prevalent, *Chlamydia trachomatis *is now the most commonly reported sexually transmitted infection in the United Kingdom [3]. Opportunistic screening of sexually active women and men under 25 years of age is being introduced in England with the aim of reducing the prevalence of chlamydia and of severe reproductive tract morbidity in women [4]. Population screening for chlamydia has become more feasible in recent years because of nucleic acid amplification tests that can be carried out reliably on non-invasively collected specimens such as urine and vulval swabs. Testing kits can be sent to large numbers of people who can collect specimens in the privacy of their own home [5,6]. Given its innovatory nature, there is as yet no substantial evidence about the psychological impact of receiving an invitation for chlamydia screening and of taking part in home testing. Qualitative research has shown that women find accessing specialist sexual health services and being diagnosed with chlamydia stigmatising [7,8] and distressing [9], so receiving an unsolicited invitation to be tested for a sexually transmitted infection may also evoke feelings of stigma and adversely impact on the recipient's sense of self worth. Research has also revealed that being tested positive for chlamydia introduces concern about the potential impact of such a diagnosis on relationships [8] and worries about future fertility [8,9]. Such anxieties could also be triggered by an invitation to be screened for chlamydia. Receiving an invitation to attend for breast screening has been reported to provoke anxiety in the recipients [10,11].

The Chlamydia Screening Studies (*ClaSS*) project was a multidisciplinary series of studies designed to investigate epidemiological, laboratory, economic, and social and psychological aspects of systematic home-based screening for chlamydia [12]. The purpose of the study reported here was to assess whether home-based screening for chlamydia had an adverse effect on levels of anxiety, depression and self-esteem in those screened and whether anxiety scores returned to pre-screening levels in those receiving negative test results.

## Methods

The rationale and methods of the *ClaSS *project have been described previously [12]. In brief, 19,773 men and women aged 16 to 39 years registered with one of 27 general practices in the Bristol and Birmingham areas were selected at random. After an interim analysis in the first four practices showed chlamydia prevalence in the older age group to be very low [12], invitations were subsequently only sent to those aged 16–25 years. They each received an invitation letter from their general practitioner followed by a study pack with information and instructions on how to provide a home-collected urine and/or vulval swab specimen to be tested for *C. trachomatis *[12]. Study packs were sent to individuals in each practice in turn between February 2001 and July 2002. Participants with negative test results were informed by letter, and those with positive results were sent an appointment to see a practice nurse at their general practice.

### Study population

As described above, the potential sampling frame was all individuals aged 16–39 years in 27 randomly selected practices. We excluded individuals selected from the first two general practices in case publicity surrounding the launch of the *ClaSS *project influenced the pre-screening measurements. We planned to measure responses in a cohort of 1000 individuals randomly sampled from all those invited to be screened, stratified by age group (16–25 and 26–39 years), sex and practice. Data were intended to be collected at three time points: one month before the invitation was posted (baseline); at the time of screening; and after receipt of a negative test result. Because of low response rates to the screening study overall, accrual of the cohort was slow and, in the last 12 study practices, we decided instead to select independent cross-sectional random samples of individuals at each of the three time points, stratified by sex and practice (all individuals now being 16–25 years old).

As part of the *ClaSS *project all those with a positive test result and a matched sample of people who tested negative were invited to take part in a case-control study. We did not send the final questionnaire for this study to people selected for the case-control study as their experiences would not have been typical of people participating in a population-based chlamydia screening programme.

Completion of the questionnaire before screening was considered implied consent. Thereafter, all those wishing to take part in the *ClaSS *project were asked to complete and sign a consent form. The South and West Multi-Centre Research Ethics Committee approved the *ClaSS *project.

### Outcome measures

We used the Hospital Anxiety and Depression Scale (HAD) [13], which has been used in a number of studies evaluating the impact of screening, as our main outcome measure. This comprises seven item subscales for anxiety and depression, each with a total score ranging from 0 to 21. We also used the Rosenburg Self Esteem Scale [14] as a further measure of the possible stigmatising effects of screening for a sexually transmitted infection. This comprises ten items with a total score range of 10 to 40.

### Sample size

Using data from a study of anxiety and breast screening, a standard deviation of 2.4 for paired differences between anxiety scores recorded at baseline and receipt of an invitation to be screened was anticipated for those in the cohort study (K. Vedhara, personal communication). For a mean difference in anxiety scores of 1, a sample size of 355 would result in a 95% confidence interval of 0.75 to 1.25 (that is a margin of error of 0.25 points on the anxiety score), taking into account the benefits of analysing within-person differences. This was considered to be an adequate level of precision. To achieve this level of response at all three time points allowing for non-response and attrition, a random sample of 1000 was initially drawn. For the cross-sectional samples we estimated that 130 participants at each time point would yield a margin of error of 0.3 standard deviations for a comparison between anxiety scores at any two time points after correcting for multiple testing.

### Statistical analysis

Analyses were undertaken using Stata [15]. The primary analysis included individuals from both the cohort and the cross-sectional samples. Mean scores for anxiety, depression and self-esteem at the three time points were compared using generalised estimating equations [16] to allow for within-individual variation in the cohort. In addition, practice was incorporated as a fixed effect and practice-specific sampling fractions were allowed for using appropriate weights in the regression analyses.

## Results

Overall, 687 people from 13 practices were invited to participate in the cohort sample and a total of 1533 people from 12 practices were invited to participate in the three cross-sectional study samples. Questionnaire reply rates to the cohort and cross-sectional approaches were very similar (Table 1). Forty-two per cent of individuals responded to the baseline questionnaire. After excluding individuals selected to be in the case-control study and those with positive results, response rates at each time point were 60% or more.

Sixty percent of the sample was female, reflecting a higher response rate amongst women (Table 2). Only 13% of the sample was aged 26 to 39 years, reflecting the age distribution of the study as a whole, as described in the Methods section. From Table 2, therefore, overall the samples are such that males were under-represented.

Moreover, from previous detailed research on the representativeness of those responding to the invitation to be screened (the time point labelled "At invitation" in this paper), it is clear that the non-response was higher for those practices with scores indicating higher levels of deprivation [17,18]. The odds ratio (OR) of response was 0.88 (95% confidence interval (CI) 0.80, 0.96; p = 0.004) for a 10-point increase in deprivation score, adjusting for age, sex and ethnicity, and taking account of sampling probability and clustering by practice [17,18]. On the other hand, after making such adjustments (especially for deprivation) there was no evidence of non-representativeness for ethnicity itself (OR = 0.98, 95% CI 0.92, 1.05; p = 0.71) [17,18]. This time point is that with the lowest overall response rate (29% from Table 1), and hence, if anything, the patterns of differential response are likely to be at their most pronounced.

The descriptive statistics for the three psychological measures according to age and sex are presented in Table 3. These descriptive statistics were very similar whether from cross sectional or cohort data. For example, the mean anxiety, depression and self esteem scores for women aged 16–25 in the cohort were 8.14, 3.63 and 7.46 respectively. The corresponding mean scores in the cross sectional data were 7.87, 3.45 and 7.56. The focus will therefore now be restricted to the analyses of all study individuals. For all three measures the scores for the majority of individuals were lower than conventional thresholds for clinical intervention, although there was considerable variation and some individuals did have levels that would cause concern. In general anxiety levels were higher amongst women but there do not appear to be differences across the age groups. Depression scores were higher in the older age group and in women. Self-esteem was lower in the younger age groups but only amongst women.

Table 4 shows, for all study individuals, the results of statistical models investigating changes in the three psychological measures over time, with full adjustment for the sampling design as well as the potential confounding effects of age and sex. In addition to the overall effects across time, the models also investigated differential time patterns according to sex and age group by considering relevant interaction terms.

**Figure 1:**
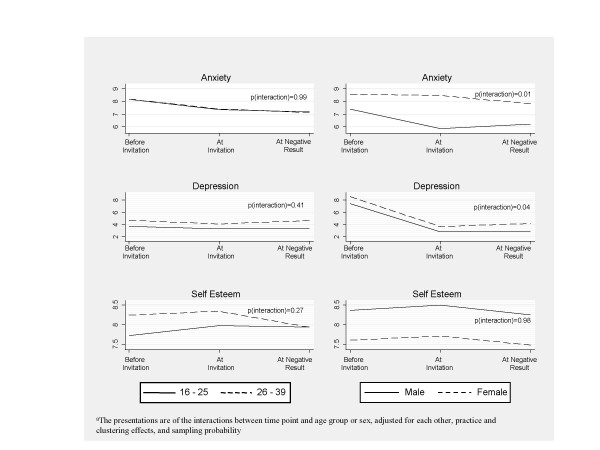
Predicted Mean Score for Three Psychological Measures According to Age, Sex and Time Point^a^.

There was a clear decrease in anxiety levels across time (overall p = 0.0049), and strong evidence that this pattern was different for men and women (p for interaction 0.012). In particular, Figure 1 shows that the decline in men's anxiety levels occurred when they submitted their test sample, whereas for women anxiety levels only declined on receipt of a negative test result. For neither sex is there any suggestion of an increase in anxiety as a result of receiving the invitation, at least amongst those responding to questionnaires. There were no clear patterns across the three time points for measures of depression and self-esteem. There is a slight suggestion of a narrowing of the different depression scores between men and women at invitation, but the evidence for this is marginal and there is no clear interpretation for this finding.

## Discussion

Most people who participate in screening programmes will receive a negative test result. From the analyses presented here, population screening for chlamydia does not seem to have a deleterious impact on the psychological well-being of those tested and found to be negative. Indeed the findings suggest that being tested for chlamydia in this way has the effect of relieving rather than creating anxiety.

### Comparisons with other studies

The findings from our study are broadly consistent with those observed for other screening programmes, which suggest that these individuals are unlikely to suffer profound or long-lasting emotional effects as a result of participating, and that the experience may even have a positive effect on their well-being. For example, a recent questionnaire-based survey of a random sample of men and women aged 15 to 29 who responded to an invitation for be screened for chlamydia by means of a home based urine sample in the Netherlands, found that 42 % felt relief at receiving a negative result and that only a small minority of those receiving a negative result remained anxious [6]. For Scottish women who participated in a series of focus groups, the wait for test results from cervical cancer screening was deemed to be an anxious period, but this was soon replaced by a sense of relief upon receipt of a normal result [19]. Sutton et al[20] assessed the psychiatric morbidity of women with negative results before and after breast cancer screening. From this study there was little evidence to suggest that breast cancer screening *per se *causes psychological morbidity. A number of studies have reported no change in anxiety and depression scores before and after screening [10,20,21], and one found a lowering of scores between baseline and screening [22]. A randomised trial of an educational intervention for women under surveillance for mild dyskaryosis detected during routine cervical screening in the UK found that the raised anxiety amongst these women was difficult to reduce [23].

Positive effects of screening of those tested negative have also been shown in other studies. Reelick et al [24] conducted a controlled before-after study of women participating in a cervical cancer-screening programme in the Netherlands and found that negative results were associated with a diminution in the scores for psychiatric disturbance at follow-up; hence screening offered a psychological benefit for some participants. Women in Bakker et al's study [25] reported that breast screening had had relatively little impact on social or physical aspects of their lives. Indeed, screening had a positive effect on certain emotional issues for at least 50% of them, including inducing feelings of reassurance that they did not have cancer, and enhancing their sense of well-being and their hopes for the future. Furthermore, Scaf-Klomp et al [26] and Gram et al [27] in their controlled studies of the psychological side effects of breast cancer screening reported less psychological morbidity post screening in those who received a negative result compared with a population sample who had not been invited to be screened. Conversely, when Stoate [28] explored whether screening can be psychologically harmful to healthy adults by assessing those invited to a coronary heart disease screening clinic, he found that levels of distress, as assessed by the General Health Questionnaire, were significantly higher after than before cardiovascular risk screening in those who received negative or low risk results, suggesting that there is a risk of causing distress when screening healthy adults.

### Study strengths and limitations

The findings reported here are based on a large, community-based chlamydia screening study that included both men and women aged 16 to 39. Although the questionnaire reply rates were not high, the response rates amongst those included in the analysis ranged from 60% to 92% across the three time points at which data were collected. This combined with the fact that the three psychometric measures used are well established, and have been the subject of rigorous psychometric testing, means that we can be confident that findings are reliable. These analyses do, however, also have a number of limitations. Low reply rates in the cohort study promoted a change of sampling method, and although we were able to fully account for the use of these two different sampling designs in the analysis, it remains the case that we were only able to analyse the psychometric data for those who responded and it is possible that the non responders had a different profile. We relied on three psychological measures that have commonly been used in this type of evaluation but it is possible that *ClaSS *had other deleterious effects not reflected in these measures. Moreover, our analyses reflect average effects of chlamydia screening and it is only possible to look in detail at the variation in peoples' experiences qualitatively [29].

## Conclusion

When screening for sexually transmitted infections there is always the potential for increasing psychological morbidity regardless of whether there are overall health benefits of screening. These findings, however, suggest that postal screening for *Chlamydia trachomatis *does not have a measurable adverse impact on psychological well-being at the population level. Indeed, individuals who took up an invitation and tested negative for chlamydia experienced a reduction in anxiety. For men this reduction occurred when they sent off a urine sample for testing; for women it came with the receipt of a negative test result. The policy implications of these findings are that unsolicited chlamydia test kits can be sent by post to population groups at risk, as part of a screening programme, because this does not appear to harm the vast majority of those screened who do not have the infection.

## Competing interests

The author(s) declare that they have no competing interests.

## Authors' contributions

RC conceived this study, wrote the first draft of this paper and led the redrafting. All other authors contributed to the redrafting of the manuscript. NM conducted all the fieldwork. ES performed the statistical analysis under the direction of RC and TP who also designed the specific methods involved. NL provided a link to the main ClaSS study which she leads.

## Pre-publication history

The pre-publication history for this paper can be accessed here:



## Figures and Tables

**Table 1:** Numbers in the study and reply and response rate, for each time period, by sampling design

	Time Period					
	Before invitation		At invitation		After receipt of negative result	
	Number sent	Number returned (%)	Number sent	Number returned (%)	Number sent	Number returned (%)
**Questionnaire reply rates**^**a**^						
						
Cohort	687	297 (43%)	680	177 (26%)	172	126 (73%)
Cross-sectional	624	250 (40%)	763	241 (32%)	146	101 (69%)
						
All	1311	547 (42%)	1443	418 (29%)	318	227 (71%)
						
**Analysis response rates**^**b**^						
						
Cohort	186	113 (61%)	186	168 (90%)	172	126 (73%)
Cross-sectional	175	105 (60%)	244	229 (94%)	146	101 (69%)
						
All	361	218 (60%)	430	397 (92%)	318	227 (71%)

a. Denominator: all individuals to whom questionnaires were posted.

Numerator: all those who replied regardless of their chlamydia test result and regardless of their inclusion in the case-control study.

b. Denominator: all individuals with a negative test result and not included in the case-control study.

Numerator: of these individuals, those who responded.

**Table 2:** Number and percentages of respondents according to age and sex for each time period

Sex	Age group	Before invitation		At invitation		After receipt of negative result		Total	
	Years	N	(%)	N	(%)	N	(%)	N	(%)
Male	16–25	64	(29)	146	(37)	76	(34)	286	(34)
	26–39	13	(6)	21	(5)	18	(8)	52	(6)
	All	77	(35)	167	(42)	94	(41)	338	(40)
									
Female	16–25	123	(56)	209	(53)	113	(50)	445	(53)
	26–39	18	(8)	21	(5)	20	(9)	59	(7)
	All	141	(65)	230	(58)	133	(59)	504	(60)
									
Total		218	(100)	397	(100)	227	(100)	842	(100)

**Table 3:** Anxiety, depression and self-esteem scores according to sex and age for all three time periods

Sex	Age group	Anxiety		Depression		Self-esteem	
	Years	Mean	(SD)	Mean	(SD)	Mean	(SD)
Male	16–25	6.52	(3.60)	3.01	(2.8)	8.57	(1.97)
	26–39	6.27	(3.38)	4.12	(3.6)	8.39	(2.34)
							
Female	16–25	8.14	(3.89)	3.63	(3.2)	7.46	(2.72)
	26–39	8.28	(3.63)	4.42	(3.2)	8.47	(2.21)
							
All	All	7.49	(3.82)	3.50	(3.1)	7.96	(2.48)

**Table 4:** Differences between mean anxiety, depression and self-esteem scores over the three time points together with p values for overall differences across time and for interactions between time point and age and sex

Score	Time Point	Difference between means	(95% CI)^1^	Difference between time points	P values Interaction with age band	Interaction with sex
Anxiety				0.0049	0.99	0.012
	Before invitation	0				
	At invitation	-0.66	(-1.23, -0.09)			
	Negative result received	-0.99	(-1.60, -0.38)			
Depression				0.25	0.41	0.041
	Before invitation	0				
	At invitation	-0.47	(-1.09, 0.15)			
	Negative result received	-0.26	(-0.91, 0.39)			
Self-esteem				0.26	0.27	0.98
	Before invitation	0				
	At invitation	0.12	(-0.26, 0.50)			
	Negative result received	-0.13	(-0.57, 0.31)			

^1 ^From model with no interaction terms. Adjusted for age band, sex, practice and clustering effects, and weighted to account for sampling probability.
